# Supplementary material for: Acetazolamide Therapy in Patients with Heart Failure: A Meta-Analysis
Source: J Clin Med. 2019 Mar 12;8(3):349. doi: 10.3390/jcm8030349 (PMC6463174; doi:10.3390/jcm8030349)
Supplement: Supplementary file 1 [file jcm-08-00349-s001.pdf]

Online supplementary data 1

Search terms for systematic review.

EMBASE: 'heart failure' AND acetazolamide: 497 articles

Databases: Ovid MEDLINE: 117 articles

1. acetazolamide.mp.
2. exp acetazolamide/
3. diamox.mp.
4. exp diamox/
5. heart failure.mp.
6. exp heart failure/
7. 1 or 2 or 3 or 4
8. 5 or 6
9. 7 and 8
10. limit 9 to human

Cochrane Databases: Heart failure AND acetazolamide: 0 article

Supplementary Materials Data

Candidate articles reviewed for full text review

-43 reviewed

-9 were included in final analysis

-4 were due to duplication of search

Hereby, list 30 article that underwent full review

-4 were either a poster or case report without search ID

1. Ali Y, Parekh AM, Rao RK, Baig MR. Metabolic alkalosis: A real danger of overdiuresis in patients with heart failure. J Int Transl Med. 2015; 3(2):120-122

2. Benge CD, Wallace J, Ooi H, Cole D, Stone W. Intravenous furosemide in the ambulatory setting for the management of a complex heart failure patient. Poster number 1207. ACC 2017

3. M. Lucas, M. Brown. Acetazolamide reduces hospital admissions and length of stay in refractory heart failure patients. Heart, Lung and Circulation abstract. 2011; 20S:S1-S155

4. T.Imiela, A. Budaj. Acetazolamide as an add-on diuretic therapy in patients with chronic heart failure exacerbations – a pilot study. Poster 4588, ESC Congress 2016

26 articles in journal articles

1. Brest AN, Likoff W. Hydrochlorothiazide in the treatment of congestive heart failure. The American journal of cardiology. 1959;3(2):144-7.

2. Brest AN, Moyer JH. Clinical pharmacology of diuretic drugs. The American journal of cardiology. 1966;17(5):626-30.

3. Cole AC. The use of diamox as a diuretic. East African medical journal. 1955;32(4):109-12.

4. Eskwith IS. The management of congestive heart failure with a free salt intake. The American journal of cardiology. 1959;3(2):184-91.

5. Goebel JA, Van Bakel AB. Rational use of diuretics in acute decompensated heart failure. Current heart failure reports. 2008;5(3):153-62.

6. Goyfman M, Zamudio P, Jang K, Chee J, Miranda C, Butler J, et al. Combined aquaretic and diuretic therapy in acute heart failure. International journal of nephrology and renovascular disease. 2017;10:129-34.

7. Hanley T, Platts MM. Acetazolamide (diamox) in the treatment of congestive heart-failure. Lancet. 1956;270(6919):357-9.

8. Hermand E, Lhuissier FJ, Larribaut J, Pichon A, Richalet JP. Ventilatory oscillations at exercise: effects of hyperoxia, hypercapnia, and acetazolamide. Physiological reports. 2015;3(6).

9. Ismail Y, Kasmikha Z, Green HL, McCullough PA. Cardio-renal syndrome type 1: epidemiology, pathophysiology, and treatment. Seminars in nephrology. 2012;32(1):18-25.

10. Jentzer JC, DeWald TA, Hernandez AF. Combination of loop diuretics with thiazide-type diuretics in heart failure. Journal of the American College of Cardiology. 2010;56(19):1527-34.

11. Knauf H, Mutschler E. Low-dose segmental blockade of the nephron rather than high-dose diuretic monotherapy. European journal of clinical pharmacology. 1993;44 Suppl 1:S63-8.

12. Knauf H, Mutschler E. Functional state of the nephron and diuretic dose-response--rationale for low-dose combination therapy. Cardiology. 1994;84 Suppl 2:18-26.

13. Knauf H, Mutschler E. Sequential nephron blockade breaks resistance to diuretics in edematous states. Journal of cardiovascular pharmacology. 1997;29(3):367-72.

14. Leiter L. Combinations of diuretics in the treatment of edema. American heart journal. 1970;80(3):422-6.

15. Massumi RA, Evans JM. Studies on the continuous use of a carbonic anhydrase inhibitor (diamox) in ambulatory patients. American heart journal. 1955;49(4):626-32.

16. Mekontso Dessap A, Roche-Campo F, Kouatchet A, Tomicic V, Beduneau G, Sonneville R, et al. Natriuretic peptide-driven fluid management during ventilator weaning: a randomized controlled trial. American journal of respiratory and critical care medicine. 2012;186(12):1256-63.

17. Moyer JH, Ford RV. Laboratory and clinical observations on ethoxzolamide (cardrase) as a diuretic agent. The American journal of cardiology. 1958;1(4):497-504.

18. Peixoto AJ, Alpern RJ. Treatment of severe metabolic alkalosis in a patient with congestive heart failure. American journal of kidney diseases : the official journal of the National Kidney Foundation. 2013;61(5):822-7.

19. Shotan A, Dacca S, Shochat M, Kazatsker M, Blondheim DS, Meisel S. Fluid overload contributing to heart failure. Nephrology, dialysis, transplantation : official publication of the European Dialysis and Transplant Association - European Renal Association. 2005;20 Suppl 7:vii24-7.

20. Suarez RM, Sr., Suarez RM, Jr., Buso R, Sabater J. [Diamox; a new non-mercurial diuretic for oral administration; clinical observations on the treatment of edema due to cardiac failure]. Boletin de la Asociacion Medica de Puerto Rico. 1953;45(10):381-6.

21. Taylor SH. Diuretic therapy in congestive heart failure. Cardiology in review. 2000;8(2):104-14.

22. Verbrugge FH, Grieten L, Mullens W. New insights into combinational drug therapy to manage congestion in heart failure. Current heart failure reports. 2014;11(1):1-9.

23. Verbrugge FH, Mullens W, Tang WH. Management of Cardio-Renal Syndrome and Diuretic Resistance. Current treatment options in cardiovascular medicine. 2016;18(2):11.

24. Verbrugge FH, Nijst P, Dupont M, Penders J, Tang WH, Mullens W. Urinary composition during decongestive treatment in heart failure with reduced ejection fraction. Circulation Heart failure. 2014;7(5):766-72.

25. Verbrugge FH, Nijst P, Dupont M, Reynders C, Penders J, Tang WH, et al. Prognostic value of glomerular filtration changes versus natriuretic response in decompensated heart failure with reduced ejection. Journal of cardiac failure. 2014;20(11):817-24.

26. Warshaw LJ. Evaluation of chlorothiazide in the diuretic therapy of congestive heart failure. The American journal of cardiology. 1959;3(2):167-79.
